# Sensory-Processing Sensitivity Is Associated with Increased Neural Entropy

**DOI:** 10.3390/e25060890

**Published:** 2023-06-02

**Authors:** Nike Walter, Nicole Meinersen-Schmidt, Patricia Kulla, Thomas Loew, Joachim Kruse, Thilo Hinterberger

**Affiliations:** 1 Department of Psychosomatic Medicine, University Hospital Regensburg, 93059 Regensburg, Germany; 2 Department for Clinical Psychology and Trauma Therapy, University of the Bundeswehr Munich, 85579 Neubiberg, Germany

**Keywords:** sensory-processing sensitivity, EEG, self-organized criticality, nonlinear dynamics, entropy

## Abstract

Background: This study aimed at answering the following research questions: (1) Does the self-reported level of sensory-processing sensitivity (SPS) correlate with complexity, or criticality features of the electroencephalogram (EEG)? (2) Are there significant EEG differences comparing individuals with high and low levels of SPS? Methods: One hundred fifteen participants were measured with 64-channel EEG during a task-free resting state. The data were analyzed using criticality theory tools (detrended fluctuation analysis, neuronal avalanche analysis) and complexity measures (sample entropy, Higuchi’s fractal dimension). Correlations with the ‘Highly Sensitive Person Scale’ (HSPS-G) scores were determined. Then, the cohort’s lowest and the highest 30% were contrasted as opposites. EEG features were compared between the two groups by applying a Wilcoxon signed-rank test. Results: During resting with eyes open, HSPS-G scores correlated significantly positively with the sample entropy and Higuchi’s fractal dimension (*Spearman’s* ρ = 0.22, *p* < 0.05). The highly sensitive group revealed higher sample entropy values (1.83 ± 0.10 vs. 1.77 ± 0.13, *p* = 0.031). The increased sample entropy in the highly sensitive group was most pronounced in the central, temporal, and parietal regions. Conclusion: For the first time, neurophysiological complexity features associated with SPS during a task-free resting state were demonstrated. Evidence is provided that neural processes differ between low- and highly-sensitive persons, whereby the latter displayed increased neural entropy. The findings support the central theoretical assumption of enhanced information processing and could be important for developing biomarkers for clinical diagnostics.

## 1. Introduction

Over the past two decades, research has explored theoretical frameworks for individual differences in the capacity to process sensory stimuli. One area that emerged during this time is sensory-processing sensitivity (SPS), which is viewed as a psychological construct comprised of perceptual sensitivity, and cognitive and emotional responses to environmental stimuli [1]. Aron and Aron initially characterized SPS as a categorical trait that reflects inter-individual differences in sensitivity to subtle stimuli, identifying those scoring high on SPS as Highly Sensitive Persons (HSP) [2]. SPS was further characterized by increased depth of information processing, enhanced environmental subtlety awareness, and ease of overstimulation [3,4,5].

To capture the degree of SPS, the Highly Sensitive Person Scale (HSPS), a 27-item questionnaire, was developed with three factors: Ease of Excitation (EOE), characterizing individuals easily overwhelmed by stimuli, Low Sensory Threshold (LST), characterizing unpleasant sensory arousal in response to external stimuli, and Aesthetic Sensitivity (AES), describing those deeply moved by arts or music [1,6,7]. Various studies have linked EOE and LST to negative emotionality, anxiety, and depression [8], whereas AES has been associated with positive emotionality, openness to experience, conscientiousness, positive affect, and self-esteem [9,10]. Approximately 20–30% of the general population is estimated to possess heightened sensory sensitivity [5,11,12]. Hitherto, SPS is mainly captured based on questionnaires or behavioral observational assessments. Despite a few conducted fMRI studies [13,14,15,16,17], little is known about the neurobiological basis of SPS, and to date, no electroencephalography (EEG) study has been carried out to determining the neurophysiological correlates of SPS.

Besides the standard method of spectral decomposition in neuroscience, advances in the field of nonlinear dynamics brought forth multiple metrics for determining the complexity of neuronal activity providing indices of information processing functions in the brain. Some of these are applicable to single time-series to quantify statistical similarity at different time scales [18,19]. Specifically, in recent years, increasing attention has been given to the hypothesis that neural dynamics might be governed by the phenomenon of self-organized criticality. This premise is based on findings of criticality hallmarks, such as scale-free distribution of neuronal avalanches and the presence of long-range temporal correlations (LRTC) [20]. In the context of SPS, this is especially compelling as critical state dynamics, representing a complex state at the edge between order and disorder [21,22], were associated with maximized input susceptibility in neuronal networks [23,24]. 

Therefore, with the purpose of investigating the neurophysiological complexity of SPS, the aim of this study was two-fold:

(1) To determine whether the self-reported level of SPS correlates either with (i) neuronal complexity determined by fractal dimension and sample entropy, and (ii) features of criticality such as a scale-free distribution of neuronal avalanches and the presence of long-range temporal correlations.
(2) To examine whether persons scoring high on the HSPS reveal differences in the above-mentioned EEG parameters during a task-free resting state compared to individuals scoring low on the HSPS.

## 2. Materials and Methods

### 2.1. Data Acquisition and Participants

The participants were recruited throughout Germany via various social networks, forums, the Research Association for sensory-processing sensitivity, and internal university invitation notifications. An amount of 30 euros was offered as an incentive to participate in the study. Psychology students received subject hours. Participation was accepted from the age of 18 years. Exclusion criteria were known epilepsy, acute self-harm, acute suicidality, and substance dependence. All 115 participants signed an informed consent before participating in the laboratory study. The laboratory surveys took place in a sound- and magnetic field-isolated cabin from 3 May–2 July 2021 on the campus of the University of the Bundeswehr in Munich. Electrophysiological data were recorded using a 72 channels QuickAmp amplifier system (BrainProducts GmbH, Munich, Germany). EEG was measured with a 64-channel ANT Waveguard electrode cap (ANT B.V., Enschede, The Netherlands) with active shielding and Ag/AgCl electrodes, which were arranged according to the international 10/10 system. Data were acquired during a task-free resting state with eyes closed and eyes open for a duration of 3 min each. Before the recording, all participants filled in the questionnaire ‘High Sensitive Person Scale’ (HSPS-G) [25]. The HSPS-G (HSP scale, original version Aron and Aron, 1997 [2]; German version Konrad and Herzberg, 2017 [25]) is a 26-item self-reported questionnaire that measures the degree of sensitivity in a 5-point Likert rating scale (“0” does not apply at all—“4” applies completely). For this purpose, the measurement instrument is divided into the subscales of *Ease of Excitation* (EOE), *Aesthetic Sensitivity* (AES), and a *Low Sensory Threshold* (LST). The HSPS-G was normed and standardized on individuals from the general population and was found to have good reliability (Cronbach’s α of 0.93 to 0.95) [25]. 

### 2.2. Data Processing 

Data were sampled at 250 Samples/s in a range from DC to 70 Hz with a notch filter at 50 Hz. After detrending the 64 EEG channels, a correction for eye movement was conducted using a linear correction algorithm [26]. Four complexity and criticality analyses were conducted as described in detail previously in Walter and Hinterberger (2022) [27]. All analyses were calculated from the mean of the time series across participants. These included a calculation of Higuchi’s fractal dimension (HFD) [28] and the sample entropy (SE) [29]. For the latter, a template length m of 2 was chosen, and the similarity criteria r was set to 0.2. To quantify long-range temporal correlations (LRTC) (i.e., the scale-invariance) in the amplitude envelope of neuronal oscillations, detrended fluctuation analysis (DFA) was applied [30]. For the assessment of critical brain dynamics the neuronal avalanche analysis was used [31]. The analysis yields two parameters: the power law scaling parameter SNZ (critical exponent) and a parameter termed SNZdiff denoting the difference between the critical exponent SNZ and the avalanche shape collapse scaling parameter. As these should be identical for brain dynamics operating in a critical regime, SNZdiff indicates the distance to the critical point used [31].

### 2.3. Statistics

Matlab (MathWorks, Natick, MA, USA) and SPSS (IBM SPSS Statistical Package 28.0, IBM Corporation, Armonk, NY, USA) were used for data analysis. To calculate correlations between the EEG features and the HSPS-G summary score as well as subscales, Spearman’s rank correlation was applied after determining that the distribution was not appropriate for parametric testing by the Shapiro–Wilk test. For this analysis, the whole cohort (*n* = 115) was considered. Then, after trichotomizing the scale, the lowest and the highest 30% of the sample were contrasted as opposites. Thus, the sample was grouped regarding the HSPS-G summary score into high sensitive persons (HSP, 78–104) and low-sensitive persons (LSP, 0–43) participants [12]. EEG features were compared between the two groups by applying an one-way analysis of variance (ANOVA). In addition, to test whether the scores are predictive of functionality, the analysis was also performed the other way around. For this, the cohort was grouped according to high and low complexity values for each parameter, respectively, by using the mean value of the complexity parameter as a cutoff. Then, HSPS-G summary scores of these groups were compared using ANOVA. Significance was set at *p* < 0.05.

## 3. Results

A total of *n* = 115 participants were recruited (mean age = 33.1 ± 13.3, 71.3% female). Detailed sociodemographic data can be found in Table 1. The first group (highly sensitive persons, HSP) consisted of *n* = 47 participants (mean age 41.75 ± 12.7 years, 24 females/23 males), with a mean HSPS-G summary score of 85.14 ± 7.7. The second group (low sensitive persons, LSP) comprised *n* = 32 participants (mean age 38.15 ± 5.1 years, 20 females/12 males) with a mean HSPS-G summary score of 22.97 ± 10.35. The groups did not differ statistically significantly regarding age (*p* = 0.869), sex (*p* = 0.649), current living situation (*p* = 0.586), education (*p* = 0.593), and job status (*p* = 0.632) (Table 1). 

### 3.1. Correlations between Sensory Processing Sensitivity, EEG Complexity, and Criticality Features

To determine whether the scores on the HSPS-G scale are significantly associated with the estimated EEG features, Spearman’s rank correlation was applied. The complexity parameter showed positive correlations between the summary score HSPS-G and MSE scale factor 1, 5, and 20, as well as HFD (*Spearman’s* ρ = 0.22, *p* < 0.05). At the subscale level, positive associations were found only with the factor EOE and SE and HFD. No significant associations were found between HSPS-G and criticality values (Table 2). During the resting state with eyes closed, the summary scores did not significantly correlate with any of the complexity or criticality values. In addition, when controlling for confounding variables, neither age, sex, current living situation, education, nor job status was found to be statistically significantly correlated with any of the complexity parameter.

### 3.2. Group Comparisons

**Figure 1:**
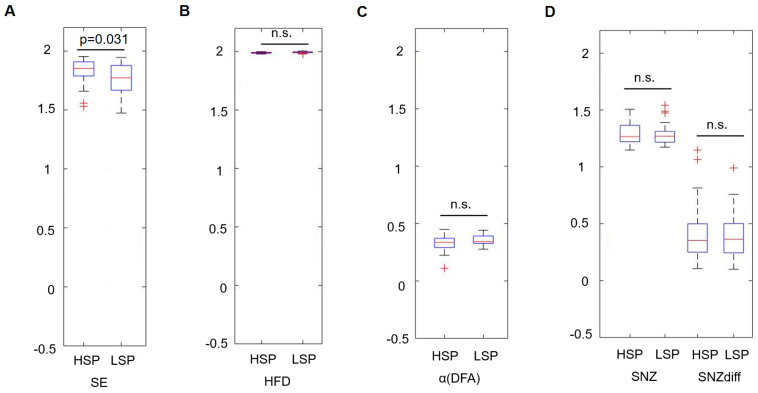
Comparison of the temporal mean of the power spectral density for each complexity parameter during a task-free resting state between the highly sensitive and the low sensitive group with ANOVA. (**A**) shows the results of the sample entropy, (**B**) HFD, (**C**) the scaling exponent resulting from the DFA, (**D**) the critical exponent SNZ, and the distance to the critical point (SNZdiff). HSP = highly sensitive person, LSP = low sensitive person, n.s. = not significant.

**Figure 2:**
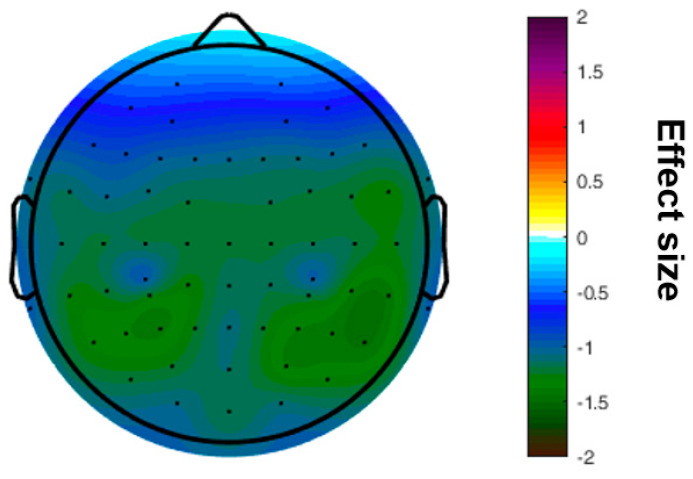
Topographical map of differences in the effect size calculated for the sample entropy comparing the groups of highly sensitive persons and low sensitive persons.

A significant difference was found for the mean SE, which was higher in the HSP group (1.83 ± 0.10 vs. 1.77 ± 0.13, *p* = 0.031) (Figure 1). The increased sample entropy in the HSP group was most pronounced in the central, temporal, and parietal regions (Figure 2). Further, it was tested, whether those participants with higher complexity values also report a higher HSPS-G summary score. This additional comparison yielded statistically significant results when the cohort was grouped according to the mean SE (HSPS-G summary score: 54.3 ± 25.4 vs. 63.2 ± 25.8, *p* = 0.037), however not for the other complexity parameter (Table 3). 

## 4. Discussion

The present study investigated electrophysiological correlates during a task-free resting state in association with the level of SPS in 115 subjects using analytical tools from criticality theory (detrended fluctuation analysis, neuronal avalanche analysis), as well as complexity measures (sample entropy and Higuchi’s fractal dimension). A major strength of the study represents the use of multiple nonlinear parameters to determine the neurophysiological signatures of SPS as it has been emphasized that each complexity measure gives additional information about the underlying data [32].

The analysis was based on the hypothesis that there might be a correlation between the level of SPS and criticality and neural complexity. While the notion that neural dynamics are governed by the phenomenon of self-organized criticality has received criticism [20], a substantial body of research emphasized the potential of critical dynamics as general indicators of healthy functioning of information processing and a surrogate measure of consciousness [33,34]. Such findings inspired the research question and the investigation whether the modulation of critical dynamics might be an underlying mechanisms of SPS. 

A few authors have also demonstrated that the amount of integrated information is largest near the critical point in network model [35,36]. Further, previous studies showed that neuronal networks operating near a critical phase transition show enhanced input sensitivity to changes in external inputs, which supports the presented premise associating criticality with heighened susceptibility [23,37]. Explanations for the phenomenon include that nodes are more excitable in a critical subpopulation and, hence, can more effectively amplify weak stimuli [38]. In addition, previous findings indicated a link between self-regulated top-down modulation of attention and a shift in the critical regime [39]. For instance, an alteration of critical dynamics in association with increased attentional load has been shown during a visuomotor cognitive finger-tapping task [40]. Further, statistically significant differences in the critical exponent were reported comparing distinct meditative states [27]. Marginally subcritical dynamics were associated with enhanced stimulus discriminability under attention [41]. However, the level of self-reported SPS did not significantly correlate with any of the criticality features and, thus, a modulation of the critical regime as the underlying mechanisms of SPS could not be confirmed in this study. 

Therefore, although the theory of SPS implies a biological foundation, the regulation of its neural basisremains a topic of investigation.

To further unreveal alterations in brain activity, the cohort was additionally grouped regarding the HSPS-G summary score into highly sensitive and low sensitive participants, and EEG features were compared. The analysis revealed a significant difference between the degree of SPS and increased complexity in terms of the sample entropy. Entropy measures are a well-established tool for the quantification of the brain’s information processing capacity [42]. Thus, this finding can be interpreted to reflect a greater depth of information processing in HSP and provide evidence for specific neurophysiological differences, especially considering that the entropy increase was only observed for the resting state with eyes open, but not for the resting state with eyes closed. This may be supported by previous studies reported higher values of entropy in experienced meditators during the practice of focused attention [27,43]. Besides constant monitoring, the focused attention meditation requires executive control in terms of detecting phases of mind wandering, where attention is directed elsewhere. Such training was shown to increase the depth of information processing and enhance the allocation of attentional resources [44,45]. 

In line with this assumption, past investigations demonstrated that HSP performed better in a visual search task, although subsequently reporting more stress compared to non-HSP. The authors explained the greater performance with higher activation of working memory in HSP [46]. Notably, significantly higher entropy values were further observed in the EEG of healthy participants performing a visual memory task [47] Moreover, it has been suggested that in HSP, the filter of the thalamus, which serves to sort out irrelevant information, considers more stimuli as relevant as in non-HSP. Consequently, environmental stimuli are perceived in finer nuances, which leads to a much wider spectrum of perceived and received stimuli but also to chronic stress experience [5,17]. This would also be in line with the entropic brain theory by Carhart Harris and colleagues, proposing that the entropy of brain activity indexes the informational richness of conscious states [48]. 

### Limitations

This study shows several limitations. First, the ad-hoc obtained sample (*n* = 115) was heterogeneous (71.3% women; 38.1% psychology students). In the HSP group, the majority of participants were female. Note, it has been suggested that, first, women score higher on the HSPS-G and, second, women identify more with the construct SPS [4,25]. As recruitment took place through the High Sensitivity Research Association, groups (https://www.hochsensibel.org, accessed on 29 May 2023) and forums for highly sensitive people, and email distribution lists of HSP coaches, it cannot be ruled out that study participants were aware of the SPS trait and the associated questionnaire, and through this knowledge, instead of their own experience, self-beliefs were reflected. Another limitation stems from the construction of the HSPS-G [25]. The scale consists exclusively of positively worded items. Thus, acquiescence tendencies on the part of the subjects cannot be excluded. This response style leads to more frequent response profiles that overestimate the presence of actual HSP in the sample. A possible solution for future research would be to invert some items. In addition, the HSPS-G is based on Aron and Aron’s original 1997 scale, which has not been modified since [2]. In the last 20 years, research interest in SPS has increased considerably, so there is a demand for an adapted measurement instrument that also increasingly captures the positive aspects of SPS [4]. Last, the question remains to what extent the HSPS-G measures SPS, as there is much overlap in content with psychopathological symptoms, e.g., from the neurotic, stress, somatoform, and affective disorder spectrums. In addition, the group division limits the generalizability of the results and the comparison with existing studies. For this reason, not only extreme group comparisons but also correlative results were reported. Finally, it should be noted that the different investigators potentially could have influenced the largely sensitive subjects. HSP can sometimes be strongly influenced by people’s moods or emotions [17]. For this reason, the investigators were extensively trained and also received detailed instructions on dealing with HSP to minimize corresponding biases. 

## 5. Conclusions

The present study demonstrated, for the first time, neurophysiological complexity associated with SPS, which could be of importance, inter alia, for the development of biomarkers for clinical diagnostics to differentiate psychopathologies and for monitoring the effectiveness of therapeutic interventions. Hereby, evidence is provided that neural processes differ between HSP and LSP. High levels of SPS were associated with statistically significant increases in the sample entropy during a task-free eyes opened resting state. The findings support the central theoretical assumption of enhanced information processing in HSP. 

## Figures and Tables

**Table 1:** Sociodemographic data (*n* = 115). After trichotomizing the Highly Sensitive Person Scale (HSPS-G), the lowest and the highest 30% of the sample were contrasted as opposites and divided into the groups high sensitive persons (HSP) and low sensitive persons (LSP).

	Total *n* (%)	HSP *n* (%)	LSP *n* (%)
	115	47	32
Age (years)	33.1 ± 13.3	41.75 ± 12.7	38.15 ± 5.1
Sex			
Male	32 (27.8)	23 (48.9)	12 (37.5)
Female	82 (71.3)	24 (51.1)	19 (59.4)
Diverse	1 (0.9)		1 (3.1)
Living situation			
Alone	25 (21.7)	10 (25.6)	5 (12.5)
With partner/family	48 (41.7)	22 (56.4)	12(30.3)
Flat-sharing community	8 (7)	4 (10.3)	2 (5.0)
Relationship yes	76 (66.1)	28 (71.8)	26 (65.0)
Education			
Secondary school certificate	13 (11.3)	9 (23.1)	2 (5.0)
Baccalaureate	45 (39.1)	8 (20.5)	21 (52.5)
Bachelor	24(20.9)	4 (10.3)	12(30)
Master	9 (7.8)	4 (10.3)	2 (5.0)
Diploma	10 (8.7)	5 (12.8)	2 (5.0)
PhD	1 (0.9)	1 (2.6)	-
Other	13 (11.3)	8 (20.5)	1 (2.5)
Job			
Self-employed	1 (9.6)	7 (17.9)	1 (2.5)
Employee	39 (33.9)	21 (53.8)	7 (17.5)
Stay at home	1 (9)	1 (2.6)	-
Student	48 (11.3)	4 (10.3)	3 (7.5)

**Table 2:** Spearman correlations of the Highly Sensitive Person Scale, complexity, and criticality parameter from the mean of the time series of the eyes open resting state across participants after averaging over channels; *n* = 115. * *p* < 0.05.

rho	HSPS-G_SUM	HSPS-G_EOE	HSPS-G_LST	HSPS-G_AES
SE	0.199 *	0.215 *	0.183	0.090
HFD	0.214 *	0.228 *	0.201	0.122
α(DFA)	−0.088	−0.088	−0.091	−0.100
SNZ	−0.050	−0.070	−0.050	0.020
SNZdiff	0.028	0.027	0.040	−0.068

HSPS-G_SUM = Summary score of the German Highly Sensitive Person Scale, HSPS-G_EOE = Subscale Ease of Excitation of the German Highly Sensitive Person Scale, HSPS-G_LST = Subscale Aesthetic Sensitivity of the German Highly Sensitive Person Scale, HSPS-G_AES = Subscale Low Sensory Threshold of the German Highly Sensitive Person Scale. SE = sample entropy, HFD = Higuchi’s fractal dimension, DFA = Detrended fluctuation analysis.

**Table 3:** Comparison of Highly Sensitive Person Scale scores in association with the EEG complexity features.

	HSPS-G Summary Score in Association with SE	HSPS-G Summary Score in Association with HFD	HSPS-G Summary Score in Association with DFA	HSPS-G Summary Score in Association with SNZ	HSPS-G Summary Score in Association with SNZdiff
Low complexity values	54.3 ± 25.4 (*n* = 49)	58.0 ± 27.6 (*n* = 78)	60.5 ± 24.8 (*n* = 42)	60.4 ± 25.9 (*n* = 57)	60.4 ± 26.8 (*n* = 63)
High complexity values	63.2 ± 25.8 (*n* = 66)	62.5 ± 23.6 (*n* = 37)	58.8 ± 27.3 (*n* = 73)	58.5 ± 26.9 (*n* = 58)	58.2 ± 26.0 (*n* = 52)
*p*-value (ANOVA)	0.037	0.197	0.747	0.695	0.650

HSPS-G_SUM = Summary score of the German Highly Sensitive Person Scale, SE = sample entropy, HFD = Higuchi’s fractal dimension, DFA = Detrended fluctuation analysis.

## Data Availability

The datasets used analyzed during the current study available from the corresponding author on reasonable request.
